# Feeding of 1-Kestose Induces Glutathione-S-Transferase Expression in Mouse Liver

**DOI:** 10.3390/foods8020069

**Published:** 2019-02-13

**Authors:** Takumi Tochio, Yuki Ueno, Yasuyuki Kitaura, Mikako Shinohara, Yoshihiro Kadota, Kanako Minoda, Yoshiharu Shimomura, Toshihiko Osawa

**Affiliations:** 1 Research and Development Center, B Food Science Co., Ltd., Aichi 478-0046, Japan; m-shinohara@bfsci.co.jp (M.S.); y-kadota@bfsci.co.jp (Y.K.); k-minoda@bfsci.co.jp (K.M.); 2 Department of Health and Nutrition, Faculty of Psychological and Physical Science, Aichi Gakuin University, Aichi 470-0195, Japan; ueno@dpc.agu.ac.jp (Y.U.); osawat@dpc.agu.ac.jp (T.O.); 3 Department of Applied Biosciences, Graduate School of Bioagricultural Sciences, Nagoya University, Nagoya 464-8601, Japan; ykitaura@agr.nagoya-u.ac.jp (Y.K.); shimo@agr.nagoya-u.ac.jp (Y.S.)

**Keywords:** prebiotics, 1-Kestose, glutathione-S-transferase (GST), antioxidative activity

## Abstract

Functional food ingredients, including prebiotics, have been increasingly developed for human health. The improvement of the human intestinal environment is one of their main targets. Fructooligosaccarides (FOS) are oligosaccharide fructans that are well studied and commercialized prebiotics. 1-Kestose, one of the components of FOS, is considered to be a key prebiotic component in FOS. However, to our knowledge, no studies have been reported on the physiological efficacy of 1-Kestose regarding its anti-oxidative activity. In the present study, we examined the effects of dietary 1-Kestose on gene expression of antioxidative enzymes in the liver, kidney and epididymal adipose tissue of mice by quantitative RT-PCR (qRT-PCR). We demonstrated that a 1-Kestose-rich diet increased mRNA and enzymatic activity levels of glutathione-S-transferase (GST) in mouse liver. These results suggest the possibility that dietary 1-Kestose as a prebiotic may enhance antioxidative activity in mice.

## 1. Introduction

The definition of prebiotics is “non-viable food components that confer a health benefit to the host associated with modulation of the microbiota” [1]. Oligosaccharides are usually used as prebiotics and are important food ingredients. Fructooligosaccharides (FOS) are widely commercialized and researched prebiotics, for which several beneficial properties have been reported. Daily intake of FOS increases levels of fecal bifidobacteria, which is associated with a trend toward a relative increase in specific immune response [2], delays the onset of senescence including learning and memory disorders in senescence-accelerated mice [3], prevention of aberrant crypt foci in mice [4], and modulation of cytokine secretion in human peripheral blood monocyte [5]. FOS consist of different ratios of 1-kestose, nystose, and fructofranosylnystose, which have 1–3 fructose monomers linked with sucrose via β2,1 glycosidic bonds [6,7]. Therefore, the key components of prebiotic activity had not been well characterized in FOS. A recent in vitro study using several oligosaccharides and lactobacilli suggested that 1-kestose content was crucial for prebiotic activity in FOS [8], and we performed in vivo studies using 1-kestose previously [9]. We examined the effects of dietary 1-kestose on cecal microbiota composition and cecal contents of short-chain fatty acids and lactate in rats. The findings indicate that dietary 1-kestose induced cecal hypertrophy and alterations in the cecal microbiota composition, including a marked increase in the cell number of *Bifidobacterium* spp. These alterations were associated with significant increases in acetate and lactate, and a marked increase in butyrate in cecal contents.

Oxidative stress is mediated by the balance between the amount of reactive oxygen species (ROS) and the activity of antioxidant systems such as antioxidants and antioxidative enzymes [10,11]. High oxidative stress occurs when there is an imbalance toward increased oxidative activity by ROS from the antioxidant (reducing) action by antioxidant substances. Many studies have reported that high oxidative stress in the body is related to many diseases such as atherosclerosis, angina pectoris, myocardial infarction, Parkinson’s disease and Alzheimer’s disease [12,13,14,15,16].

Some studies have shown a link between prebiotics and antioxidation [17]. It has been shown that butyrate, one of short chain fatty acid produced as bacteria metabolites, increased GST expression in the human colon cells [18], suggesting that antioxidative activity is associated with the components produced in the intestine. On the other hand, no studies have been conducted to examine the in vivo effects of 1-Kestose diet on the antioxidative activity in animals. Thus, the purpose of this study was to clarify the effects of 1-Kestose using mice.

## 2. Materials and Methods

### 2.1. Animal Experiments

All procedures for animal experiments in the present study were approved by the Animal Care Committee of the Graduate School of Bioagricultural Sciences, Nagoya University. 1-Kestose (purity > 98%) was provided by B Food Science Co., Ltd. (Aichi, Japan). Experimental conditions were referred to the previous study using rats [9]. Namely, experimental diets were prepared in a pellet form by CLEA Japan (Tokyo, Japan). The composition of the control diet was based on the AIN-93G diet, and sucrose in the diet was replaced with the same amount of 1-Kestose to prepare the 1-Kestose diet at 5.0% (Table 1).

Ten male ICR mice aged 8 weeks were obtained from Japan SLC (Hamamatsu, Japan), and individually housed in cages in a conventional animal room with a controlled temperature (23 ± 1 °C) and 12-h light-dark cycle (lights on at 8:00 a.m.). After acclimatization to the animal room for 1 week, the mice were randomly allocated to two groups (*n* = 5 each): control diet and 1-Kestose diet groups. Mice in each group were provided free access water and the corresponding experimental diets for 4 weeks. Food intake and body weight were recorded once a week. On the final day of the experiment, mice were sacrificed under anesthesia with isoflurane, and blood samples were obtained from the posterior vena cava with a syringe to prepare serum. Subsequently, the liver, kidney and epididymal adipose tissue were harvested and divided into two samples. One tissue sample was frozen in liquid nitrogen to measure glutathione-S-transferase (GST) activity, and the other was immersed in RNAlater (Thermo Fisher Scientific Inc., Waltham, MA, USA) for extraction of total RNA. Serum and tissues were stored at −80 °C until analyses.

### 2.2. Analysis for Blood Components

Concentrations of serum triglycerides (TG), total cholesterol (TC) and free fatty acids (FFA) were measured using the respective kits purchased from Wako Pure Chemical Industries, Ltd. (Osaka, Japan).

### 2.3. Preparation of Total RNA

Approximately 30 mg of liver, kidney and epididymal adipose tissue immersed in RNAlater were used to prepare total RNA. Extraction and purification of total RNA from tissues was carried out using a kit purchased from Macherey-Nagel (Düren, Germany). Purified total RNA was diluted in RNase-free water and used for quantitative real-time-PCR (qRT-PCR).

### 2.4. Microarray Analyses

Microarray analyses were performed using a mouse DNA chip for oxidative stress and anti-aging. Briefly, single and double-stranded cDNA were synthesized from the total RNA extracted from the tissues using the Superscript Choice System (Life Technologies, Carlsbad, CA, USA) with a T7-dT24 primer. Biotinylated cDNA was synthesized using the BioArray High Yield RNA Transcriptional Labeling Kit (Enzo Diagnostics, Farmingdale, NY, USA). After purification of the cDNA using the RNeasy Mini Kit (QIAGEN, Tokyo, Japan), cDNA was fragmented by heating at 94 °C for 35 min, then an aliquot of the obtained fragmented cDNA was hybridized to the mouse DNA chip for oxidative stress and anti-aging (Genopal, Mitsubishi Rayon CO., Ltd., Tokyo, Japan). After hybridization, the fluorescent signals were measured using the Biochip Reader (Yokogawa Electric Co., Tokyo, Japan).

### 2.5. Quantification of the Gene Expression Level Using qRT-PCR

Total RNA samples extracted from liver, kidney and epididymal adipose tissue were reverse transcribed to cDNA using the ReverTra Ace qPCR RT kit (Toyobo, Osaka, Japan). Quantification of gene expression was carried out using the qRT-PCR system (Thermal Cycler Dice Real Time System, Takara Bio Inc., Shiga, Japan). Amplification was performed in a final volume of 25 µL containing 50 ng of cDNA, optimized specific primers (Table 2) and probes (TaqMan Gene Expression Assays, Applied Biosystems, Waltham, MA, USA), and TaqMan Universal PCR Master Mix reagents (Applied Biosystems) according to the manufacturer’s directions. Results are expressed as the fold increase relative to the controls after normalization using the β-actin gene expression level.

### 2.6. Hepatic GST Activity Assay

Hepatic GST activity was measured using the SensoLyte^®^ GST Activity Assay Fluorimetric Kit (Anaspec, Fremont, CA, USA) according to the manufacturer’s instructions. Frozen liver samples (30 mg) powdered under liquid nitrogen were used for the assay.

### 2.7. Statistics Analysis

All values are expressed as mean ± standard error (SE). Data were analyzed statistically using the Student’s *t*-test. Differences were considered significant at *p* < 0.05.

## 3. Results

### 3.1. Body Weight, Food Intake and Tissue Weights

Body weight and food intake were not affected by intake of 1-Kestose diet (Table 3). The weights of the liver, kidney and epididymal adipose tissue were not altered by 1-Kestose diet, but the cecum weight and content were significantly higher in the 1-Kestose group than in the control group (Table 3).

### 3.2. Concentrations of Blood Components

Concentrations of serum TG, TC and FFA were measured. The serum TC concentration was significantly lower in the 1-Kestose group than in the control group, but the concentrations of TG and FFA were not affected by the 1-Kestose diet (Table 3).

### 3.3. Effects of Dietary Intake of 1-Kestose on Gene Expression Related to Oxidative Stress in the Tissues

First, we investigated the effects of intake of 1-Kestose diet on gene expression related to antioxidation in the liver. The number of genes with >1.5-fold higher expression in the 1-Kestose group than in the control group was three, and that for the opposite case, lower (<0.5-fold) in the 1-Kestose group than in the control group, was five. These genes are listed in Table 4.

**Figure 1:**
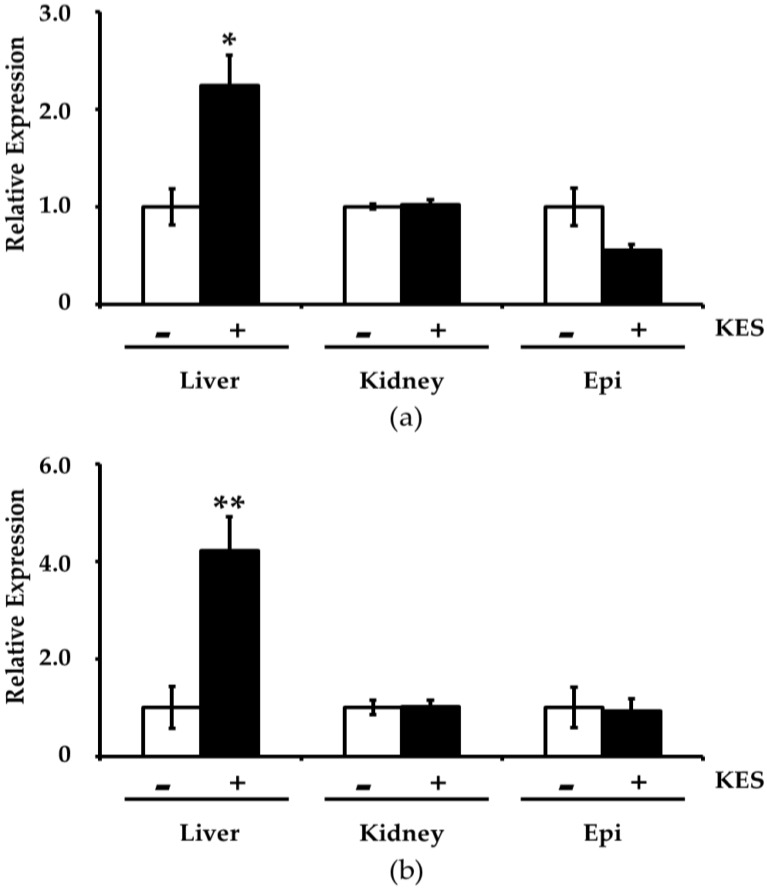
The expression of (**a**) Gstp1 and (**b**) Gsta4 in the liver, kidney and epididymal adipose tissue (Epi) was measured by real-time PCR. β-actin was used as the internal control. The relative changes were determined by the 2^−ΔΔCt^ method. KES (−): control diet group, KES (+): 1-Kestose diet group, * *p* < 0.05, ** *p* < 0.01 vs. KES (−) value by Student’s *t*-test.

Next, we quantified gene expression related to antioxidation (i.e., Gstp1 and Gsta4) in the liver, kidney and epididymal adipose tissue by qRT-PCR. Hepatic gene expression of Gstp1 and Gsta4 was higher in the 1-Kestose group than in the control group (Figure 1). On the other hand, expression of these genes in the kidney and epididymal adipose tissue was not altered by intake of 1-Kestose diet (Figure 1).

### 3.4. Hepatic GST Activity

**Figure 2:**
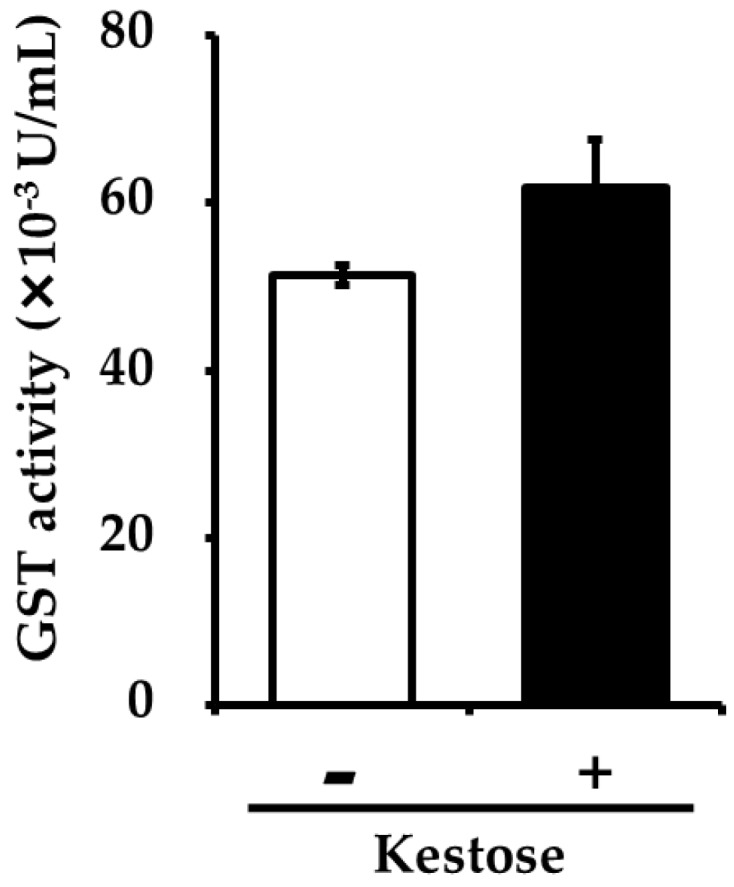
GST activity in the liver was measured using the SensoLyte^®^ GST Activity Assay Fluorimetric Kit. KES (−): control diet group, KES (+): 1-Kestose diet group.

In order to examine the effects of higher expression of hepatic Gstp1 and Gsta4 on hepatic GST activity in the 1-Kestose group, we measured the GST activity in the liver of both groups of mice. The hepatic GST activity in the liver tended to be higher, but statistically not significant, in the 1-Kestose group compared to the control group (Figure 2).

## 4. Discussion

Recent studies have suggested that the intestinal microbiota is closely linked with the development of allergies, gut chronic disorders and metabolic syndrome [19,20,21]. As diet greatly impacts the development of the well-balanced microbiota, functional food ingredients, including prebiotic oligosaccharides, which can promote the growth of gut beneficial commensals without digestion by the host, are promising for the prevention of such disorders. However, no study has been reported clarifying the effects of prebiotics on antioxidative enzymes. 

In this study, we demonstrated that feeding of 1-Kestose diet induced up-regulated gene expressions of two enzymes (Gstp1 and Gsta4) that belong to the antioxidative family and induced the GST activity in the liver of mice. These results suggested that the up-regulation of GST activity by 1-Kestose diet was induced Gsta4 and Gstp1. However, GST is an antioxidative enzyme that has several families such as Alpha (Gsta), Mu (Gstm), Pi (Gstp), Theta (Gstt), Omega (Gsto) and Zeta (Gstz), and it is possible that these enzymes involved in the up-regulation of GST as well. 1-Kestose has been reported as a prebiotic with several beneficial effects such as promotion of the growth of bifidobacteria [2]. Fermentation products of the gut microbiota are not only used as the energy sources of colonic mucosal tissue, but in some cases, are absorbed into the liver through the portal vein and then transported to other organs through the bloodstream for use. As GST expression has been reported to be induced by butyrate in human colon cells [18], we considered that up-regulation of GST in the liver by 1-Kestose feeding may be related to the increased butyrate levels in the intestine. However, further studies are needed to clarify the mechanisms.

In the present study, we demonstrated that feeding of the 1-Kestose diet increased weights of the cecum and cecal contents, likely due to the fermentation of 1-Kestose in the cecum. Similar phenomena have been found in a previous study in rats [9]. Therefore, the findings suggest that dietary 1-Kestose induced cecal hypertrophy and alterations in the cecal microbiota composition in association with the increases in short-chain fatty acids in cecal contents of mice.

## 5. Conclusions

The present study demonstrated that a 1-Kestose-rich diet induced GST expression in the liver. These findings may reveal that dietary 1-Kestose as a prebiotic has a potential to enhance antioxidative activity in the host animal, thereby reducing oxidative stress. However, the present study determined only the expression and the activity of GST, but not any direct makers of oxidative stress such as lipid peroxidation and the ratio of GST/GSST. Further studies are needed in experimental animals and humans to clarify the impact of dietary 1-Kestose on oxidative stress-related diseases.

## Figures and Tables

**Table 1:** Experimental diets.

Ingredient	Control Diet	1-Kestose Diet
	(g/100 g Diet)	
Corn starch	39.7	39.7
Maltodextrin 10	13.2	13.2
Sucrose	10.0	5.0
Casein	20.0	20.0
Soybean oil	7.0	7.0
Cellulose	5.0	5.0
Mineral mix	3.5	3.5
Vitamin mix	1.0	1.0
L-Cysteine	0.3	0.3
Choline bitartrate	0.3	0.3
1-Kestose	0	5.0
Total	100.0	100.0

**Table 2:** Oligonucleotides used in this study.

Target	GenBank Acc	Oligonucleotide Sequence
β-Actin	NM007393.4	CATCCGTAAAGACCTCTATGCCAAC
		ATGGAGCCACCGATCCACA
Gstp1	NM_0103541.1	CGGCAAATATGTCACCCTCATCTA
		TCTGGGACAGCAGGGTCTCA
Gsta4	NM_010357.3	TGACACAGACCAGGGCCATC
		ATCAGGTCCTGGGTGCCATC

**Table 3:** Body weight, food intake, organ weight and serum biochemistry.

	KES (−)	KES (+)
	Means ± SE	
Body weight (g)	33.7 ± 1.4	32.0 ± 1.3
Food intake (g/day)	3.30 ± 0.09	3.53 ± 0.12
Liver (g)	1.45 ± 0.09	1.31 ± 0.08
Kidney (g)	0.32 ± 0.01	0.31 ± 0.01
Epidermal adipose tissue (g)	1.52 ± 0.14	1.23 ± 0.16
Cecum (g)	0.09 ± 0.00	0.16 ± 0.01 *
Cecum content (g)	0.27 ± 0.02	0.43 ± 0.03 *
Serum triglycerides (mg/dL)	155 ± 23	133 ± 13
Serum total cholesterol (mg/dL)	155 ± 1	138 ± 4
Serum free fatty acids (mEq/L)	0.57 ± 0.01	0.51 ± 0.02

KES (−): control diet group, KES (+): 1-Kestose diet group, * *p* < 0.05 vs. KES (−) value.

**Table 4:** Gene expression ratio of KES (+)/KES (−).

Gene	Expression Ratio KES (+)/KES (−)
Gstp1 (glutathione S-transferase pi 1)	2.13
Tgfb3 (transforming growth factor beta 3)	1.61
Gsta4 (glutathione S-transferase alpha 4)	1.50
Cyp4a10 (cytochrome P450, family 4, subfamily a, polypeptide 10)	0.75
Srebf1 (sterol regulatory element-binding transcription factor 1)	0.75
Col18a1 (collagen type XVIII alpha 1 chain)	0.70
Cyp4a12a (cytochrome P450, family 4, subfamily a, polypeptide 12a)	0.70
Ldlr (low-density lipoprotein receptor)	0.69
Keap1 (Kelch-like ECH-associated protein 1)	0.69
Gck (glucokinase)	0.69
Gstt3 (glutathione S-transferase theta 3)	0.64
Fasn (fatty acid synthase)	0.47
Mt3 (metallothionein 3)	0.45
Apoa4 (apolipoprotein A4)	0.40
Cyp7a1 (cytochrome P450, family 7, subfamily a, polypeptide 1)	0.34
Mt1 (metallothionein 1)	0.28

KES (−): control diet group, KES (+): 1-Kestose diet group.
